# Breast cancer in women in Gaza: qualitative study of women’s expectations and experiences of diagnosis and management

**DOI:** 10.3332/ecancer.2025.1889

**Published:** 2025-04-10

**Authors:** Shaymaa AlWaheidi, Richard Sullivan, Elizabeth A Davies

**Affiliations:** 1 Centre for Cancer, Society & Public Health, King’s College London, London SE1 9RT, UK; 2 Institute of Cancer Policy, King’s Health Partners Comprehensive Cancer Centre, King’s College London, London SE1 9RT, UK

**Keywords:** breast cancer, Gaza, cancer care in conflict, Palestinian women

## Abstract

**Objective:**

This study aimed to explore women’s awareness of breast cancer and its symptoms, their experiences of accessing healthcare, diagnosis and treatment, and their fears about the stigma associated with the disease in Gaza.

**Methods:**

Semi-structured face-to-face in-depth interviews were conducted with a purposive sample of 20 women diagnosed with breast cancer in 2017 and 2018. Interviews were conducted between 2018 and 2021, before the current conflict of 2023–25. They were transcribed verbatim, translated from Arabic to English, uploaded into NVivo™ 12 computer software package and analysed using the Framework approach to identify key themes.

**Results:**

Most women presented early with their breast symptoms, but around one half identified delayed diagnosis as a major contributor to subsequent delays in treatment. Despite having relatively good experiences with their surgeons, most were frustrated by a lack of communication between them and their oncologists. Nearly all women expressed feelings of embarrassment about being seen and examined by male clinicians. Many women reported a lack of prompt and effective treatment such as Herceptin and Tamoxifen as the main barrier to cancer care, and this was also true for those who received or should have received Goserelin or radiotherapy. Women agreed that obtaining exit permits from the Israeli authorities to receive necessary treatments outside Gaza was problematic. In addition, the relationship of many married women with their husbands was negatively affected by their diagnosis.

**Conclusion:**

Women’s accounts demonstrate the need during 2017–21 for clear early detection guidelines for breast cancer to shorten the pre-diagnostic journey, improve referral pathways, build up diagnosis and histopathology capacity to reduce diagnostic delays in Gaza. However, the 2023–25 conflict has led to repeated internal displacements, the destruction of hospitals and clinical networks, and border closures has done little to help improve services and outcomes for these women. Much more work needs to be done to restore these fundamental services for women with breast cancer caught in this conflict.

## Introduction

In Gaza, there is a belief that fear, embarrassment and fatalism about breast cancer are major cultural barriers that delay a woman’s decision to seek help with breast symptoms. There is also evidence suggesting a pervasive lack of basic treatment modalities for cancer care, which has resulted in poor outcomes. Findings from our companion study reported a 1-year crude survival rate of 95% for women diagnosed with breast cancer (*N* = 524) in 2017 and 2018 [1]. These survival rates are in line with estimates from high-income countries such as the UK where the probability of surviving 1 year after diagnosis is 96% [2]. However, the same study found that more than half of all the cases were diagnosed at stage III or stage IV of breast cancer, indicating either later presentation, a reluctance to seek medical care or diagnostic delay [3, 4].

There is very little qualitative research documenting the richness and complexities of experiences of accessing cancer care in Gaza [5, 6]. This study therefore aimed to explore, using in-depth interviews. women’s awareness of breast cancer and its symptoms, their experiences of accessing healthcare, diagnosis and treatment, and their fears about the stigma associated with the disease in Gaza.

## Materials and methods

### Eligibility criteria

Inclusion criteria for the study were women living in Gaza and diagnosed with breast cancer in 2017 or 2018. Women who were unable to cope either physically or emotionally with the research process were excluded. The exclusion criteria also included cognitive problems affecting women’s memory or communication skills which meant they could not give valid consent.

### Data collection methods

At the time of the study, no official statistics showing the incident cases of breast cancer in Gaza were available for the years 2017 or 2018, which could have been used for the sampling frame. In addition, the two cancer hospitals in Gaza did not have electronic cancer databases or registries, but instead maintained all their cancer records in paper format. The only feasible and relevant sampling method was therefore to approach and recruit women with breast cancer through gatekeepers. The gatekeepers selected 20 potentially eligible patients who had shown an interest in taking part. All the women approached by the lead researcher [Shaymaa AlWaheidi (SAW)] agreed to take part and were invited for an interview at a time and place of their choice. The first in-person interviews took place between 1 July 2018 and 31 August 2018. The second interviews took place in 2021 with all except three women. One died before the interview; the second’s health had deteriorated with her tumour progressed to stage IV; and the third, out of desperation, took the opportunity of an exit permit for radiotherapy to leave Gaza. Interviews lasted between 40 minutes and 1 hour and were audio-recorded and stored securely.

### Analysis methods

Pseudonyms were given to all the participants to provide complete anonymity. The interviews were translated and transcribed and uploaded into NVivo™ 12 computer software package for analysis. To identify meaningful concepts corresponding to the research questions, SAW read each transcript, line by line and analysed the interviews using the principles of Framework Analysis [7]. The key themes and examples were discussed throughout the analysis with Elizabeth Davies (ED) and then shared and discussed with Richard Sullivan (RS).

## Results

### Participants

Demographic characteristics: 17 women were married at the time of the interview, one was single, one was widowed and one was divorced, and all but one had one or more children. Most women (*n* = 16) were housewives, three were employed and one was an unemployed graduate. Seven women had a family history of breast and/or ovarian cancer (Table 1).

Breast cancer diagnosis and treatment: Eleven women were diagnosed with disease stage II, seven at stage III, one at stage IV and one at stage I. Just over one half (*n* = 11) had undergone a lumpectomy, eight had undergone a modified radical mastectomy and one had undergone modified radical mastectomy after a lumpectomy. Of the nine women who had undergone modified radical mastectomy, none had breast reconstructive surgery. Most of the women had also undergone adjuvant chemotherapy (*n* = 17), tamoxifen (*n* = 18) and radiotherapy (*n* = 14). Radiotherapy was planned for four other women, but the permit for one was refused many times, two were still waiting for permission at the time of the interview and one did not attend for her planned treatment.

Three main themes and nine sub-themes emerged from the rich information in the interviews (Table 2). In this paper, the three themes and only five sub-themes are described as these offer the strongest implications for future research and clinical practice.

### Theme one – the journey of diagnosis

#### Initial misdiagnosis and the resulting anger caused by delays

All the women presented to the healthcare system after experiencing symptoms of breast cancer because they realised that these symptoms were not normal. 17 of the 20 women sought prompt diagnosis when they noticed these changes in their breasts, even though some of them felt anxious, embarrassed or worried about what a doctor might find. Despite prompt consultation by 17 women, for 12 there was a delay between their presenting and the confirmation of a clinical diagnosis of cancer due to issues of misdiagnosis. Dina, for example, explains:

*“When I went to the clinic… they told me that it was because I stopped breastfeeding and that it was normal… She [the doctor] told me to just massage it more... So, I did that… It was useless. I told the doctor that it didn’t work… But she didn’t care.” **Dina,*** (lines: 30-41).

After receiving false reassurances and unreasonable delays in obtaining diagnostic confirmation, these women were not able to trust the healthcare system in Gaza. Hanem thought this was the end for her:

*“I used to come, and they would tell me it was inflammation, and it would go, and not to worry… Now that’s it. Now I’m done.” **Hanem***, (lines: 32-96).

#### Receiving, tolerating and sharing the bad news

The common view among all the women was that in their community the word cancer is dreaded as a ‘death sentence’. Fatalistic and social perceptions in the framing of cancer in conversations are reflected in the language and the metaphors that women used to refer to the disease:

*“Gaza is not inhabitable let alone good for people with cancer.” **Niveen,*** (line: 247).

*“I mean the war and stuff... like I told you, it’s like the end of the world here in Gaza.” **Nadeen,*** (lines: 536-538).

Most women did not want to divulge their diagnosis, and many were uncomfortable when they discovered that some people heard the news even though they had not wanted it to spread throughout the community. The way women talked about the discovery that they had breast cancer, tolerating the shock and sharing the news with their families, vividly captures the mindset of women in this society who, because of adverse socio-cultural perceptions, keep quiet about their cancer. Tragically this unfortunate socio-cultural perception of cancer was compounded by the initial misdiagnosis of many women as described above, by the issues of unsupportive husbands and having to talk to a male doctor about their diagnosis. This will be explored in the next theme.

### Theme two – the relationship between women and the two men in their lives: the husband and the clinician

#### Marital relationships

Parents and siblings played a major role in providing women with financial and emotional support – but this situation was different when it came to the husbands of the 17 married women. While six women described how their husbands had offered a lot of practical support, ten women talked about how they had not received any support from their husbands after their diagnosis. Ola’s experience was typical of these women:

*“He treats me in a shallow way… To the point that recently he said to me go to your parents because they should have me… We sleep in different rooms these days. I feel sorry for my children… Also, I’m sick of the bandana. I have it on day and night.” **Ola,*** (lines: 289-708).

These women had a mixture of feelings of loneliness, rejection, being left out and in many cases of helplessness. They mentioned that physical changes such as surgery and hair loss due to chemotherapy represented a major challenge for their relationships and for the closeness they needed from and wanted to have with their husbands:

*“I have a hat on all day long. I mean my husband tells me to not let the kids see me without it. He tells me: I don’t like it when you keep telling me you’re sick. I mean I’ve had the operation seven months ago... My husband hasn’t seen me since then… May Allah help us.” **Karima,*** (lines: 255-677).

Another possible explanation for the distance that separated the husbands from their wives could be socio-cultural and socio-political factors. Most women in the study mentioned that in addition to having to cope with their wives’ new condition, their husbands had to cope with their own issues such as unemployment and low socio-economic status.

#### Being treated by a male doctor

All women felt uncomfortable and embarrassed about being seen and examined by a male doctor. They had all hoped that a female surgeon and oncologist would be available. Some of the women questioned themselves about whether being seen by a male doctor/surgeon was acceptable in their religion and whether God would forgive them. Feeling embarrassed and uncomfortable with male oncologists was compounded by other factors expressed by many women such as lack of privacy in crowded hospitals, rushed appointments because of the oncologists’ busy schedules and not enough curtained chemotherapy beds at the hospital. These factors are discussed in more detail in the next sub-theme.

Although Gaza is a very conservative city and the healthcare system in Gaza is a culturally tailored one, some women felt that the male doctors they had seen did not appreciate why women do not want to be examined by a man. In addition to a shortage of female doctors available in Gaza, some women said that the modesty of the patient did not seem to be of importance to the doctors. Nadeen said:

*“I asked her [the nurse] to cover me when they get me out of the operation. I begged her… They make you wear this thing and it’s seethrough.” **Nadeen,*** (lines: 339-341).

But for women, their modesty was not just a matter of being covered up or of wearing specific clothing:

*“Every day while I was at the hospital, a group of doctors would come with nearly 10 other interns, so it was very embarrassing. You feel like you’re for show. It was embarrassing.” **Dina,*** (lines: 505-508).

*“I’m a show for 10 doctors who come and look at my cut. All the supervisors of the surgery section… Yes, you’re a doctor, respectful, and I get that but the doctor who did the surgery is enough for me.” **Marwa,*** (lines: 596-599).

### Theme three – women’s experiences of breast cancer treatment

#### Unmet expectations for treatment

Despite the poor financial situation of almost all women in this study, more (*n* = 12) underwent surgery at a non-government than at a government hospital (*n* = 8). For these women, this would not have been possible without the financial support that they received from many sources, such as families and friends, donations and charitable organisations. Perceived poor quality of cancer care and delayed surgery appointments in government-run hospitals were the two main reasons mentioned by some women when they were asked about why they opted to undergo surgery at a non-government hospital.

Most women received their chemotherapy cycles according to their treatment plans, and only two had their chemotherapy cycles delayed because of the unavailability of Taxotere (docetaxel) at government hospitals when they attended. Some women also said that the immunity treatments needed in between chemotherapy cycles as well as the hormonal blocker Goserelin (Zoladex injections) were not available most of the time at government hospitals.

Those women were worried that such delays would negatively affect their treatment outcomes, and they, therefore, sought non-government initiatives and charity organisations to help them to buy the Zoladex injections that they needed but could not afford to buy for themselves. The patient support initiatives helped some of the women to avoid travelling to the West Bank and being quarantined when they got there. For example, Zeinab had been receiving Zoladex for several months at Augusta Victoria Hospital in East Jerusalem. The non-government patient support groups also helped to cover a few women’s transport fees to Jerusalem for radiotherapy. This was very important considering the poor financial situation of most women in the study. In the follow-up interview, Zeinab revealed that she had cancelled her appointments in Jerusalem because she feared that upon returning to Gaza, she would be sent to a quarantine centre for 21 days – and thus be kept away from her children. Zeinab would not have had to make such a decision had Zoladex been available in Gaza [8].

In addition to issues of poor quality of cancer diagnosis and its treatment in Gaza, the worry about being denied or delayed exit permits by the Israelis makes this treatment ‘journey’ almost unbearable. Fadwa, a 33-year-old unmarried woman, recounted that she wanted her father to be with her during her treatment in Jerusalem but filled in her mother’s name as her companion in the referral application form because she feared that the Israelis would reject her father’s application for an exit permit.

Ola submitted three referral applications – two of which were denied by the Israeli authorities, allegedly on security grounds and one which was delayed with no definitive response being received by the date of her hospital appointment. She then applied to go to Egypt, but it was too late for her because due to the COVID crisis, the borders were closed for a long time and, in the end, Ola did not receive any of her radiotherapy:

*“The last two attempts I had rejections... I used to put the name of my sister-in-law but then I thought maybe it’s because of her. So, I put the name of my mother-in-law. I mean my mother-in-law has been through before. She is 64. They rejected me again… the security rejection has really disturbed me. I applied for Egypt by the way... The radio’s time was over.” **Ola,*** (lines: 159-408).

Ola felt that she was being tortured:

*“I wish that the radiotherapy treatment wasn’t such a torture. If only they could collect donations to get this device here and bring the specialists to operate it.” **Ola,*** (lines: 615-616).

There is only one site at which radiotherapy is available for the whole of the Palestinian people which is the Augusta Victoria Hospital in East Jerusalem. There were mixed views regarding the quality of cancer care received in East Jerusalem, and all the women wished that radiotherapy were available in Gaza so that they could receive their treatment on time.

## Discussion

### Summary of main findings

Most women in this study of Gaza presented their symptoms early, but around one half identified delayed diagnosis of breast cancer as a major contributor to delays in their treatment. Apart from the difficulties of communication, nearly all women expressed feelings of embarrassment about being seen and examined by a male clinician. Even in a secular society this might be problematic, but in a religiously dominated society, this is a cause of additional distress for women already emotionally upset.

Furthermore, women felt that the medical treatment they received was substandard. Due to a lack of forthcoming information, advice and consideration, the women turned to the internet for information and sought advice from other women with breast cancer. Many women reported a lack of prompt and effective treatment as the main barrier to their cancer care, and this was especially true for those who received or should have received Goserelin or radiotherapy. All the women wished that radiotherapy was available in Gaza so that they could receive their treatment on time and avoid the need for long journeys across borders and having to leave families behind. It would also reduce the costs involved and the pressure on the radiotherapy service in East Jerusalem, which cannot cater to all the cancer patients from the whole of the Palestinian territory.

The cost of surgery in private hospitals was a barrier for most of the women. However, many did manage to borrow money from their relatives for private treatments, or else sought financial help from charitable organisation to avoid the long waiting lists for surgery in government hospitals. In addition, the relationship of many married women with their husbands was affected negatively by their diagnosis, and some expressed the need to share their stories with someone else.

Many strategies were employed by these women to cope with, or to avoid their distressing experiences. Examples of these were the non-disclosure of the diagnosis and faith in ‘Allah’ displayed by all the women in this study. Despite this, women could not escape experiencing feelings of low self-esteem due to the physical changes they had to endure, especially breast removal and hair loss during chemotherapy. Most if not all the time they wore wigs and/or breast prostheses to try to improve their looks in front of their families, and to help them to feel a bit better about themselves.

### Strengths and weaknesses of the study

This study is the first to use in-depth semi-structured interviews in Gaza to explore women’s awareness of breast cancer and its symptoms, their experiences of accessing healthcare and the diagnosis and treatments involved, and their fears about the associated stigma. Other research studies involving women with breast cancer have used face-to-face and/or self-administered structured questionnaires to address research questions mainly regarding the risk factors associated with breast cancer, early detection and screening programmes. By contrast, this study considered the multifaceted barriers to breast cancer diagnosis and treatment including individual and socio-cultural factors.

The most effective use of the limited resources of this study was achieved by relying on the experience and judgement of the gatekeepers to select women whose cases would be rich in new information. The decision to purposively sample women with breast cancer guaranteed a sufficient level of conceptual depth by focussing on women who were physically capable of contributing to in-depth interviews and to include those who had experienced different phases of their treatment journeys in different ways. However, this approach tended to exclude older women, and those with advanced stages of breast cancer because the gatekeepers judged they would not be able to cope with the interviews and the follow-up visits that would be required. This approach could have also led to selection bias as a potential reason for not knowing the experiences and needs of excluded patients. Also, the mean age at diagnosis is 7 years younger than that of women in Gaza due to this selection bias.

### Future implications

#### Barriers to early diagnosis

Fear, embarrassment and fatalism about breast cancer are major cultural barriers in the occupied Palestinian territory and in other Arab countries, and these factors influence women’s decision to seek diagnosis [9–12]. In this study, most of the women interviewed sought early presentation after noticing a change in their breasts. A possible explanation for this is increased breast awareness among women in recent years, mainly because of the increase in cancer awareness initiatives and patient advocacy groups in the country. This was evident in the interviews and was also found in Baloushah’s study, where most participants (90%, 352/390) showed a high level of awareness of breast cancer and its symptoms [13].

Because all the women in this study were treated by male oncologists and cancer surgeons, stigma and embarrassment issues appeared once the diagnoses had been confirmed. Supporting and facilitating the access of women to education in the main diagnostic and surgical specialities as well to other specialities and training programmes in nursing, palliative care and psychology could improve the gender balance and the experiences of women as a result.

While patient-mediated factors are still important in the delayed diagnosis of breast cancer, healthcare system barriers presented significant obstacles to prompt diagnosis in this study – even when women had the knowledge and attitudes to seek early diagnosis. Consistent with this, AlShiekh’s study found that one half of the women with breast cancer in Gaza were referred for imaging after many consultations, and this was also classified as a delay in diagnosis [14]. It is still important however to fund more public health programmes and patient advocacy groups, using appropriate health communication tools, to modify women’s attitudes toward breast cancer and to encourage women to seek prompt diagnosis when they notice any change in their breasts. Such groups should address cultural barriers and modify husbands’ negative beliefs and views about women diagnosed with breast cancer. If women are diagnosed with late disease, then, to promote social wellbeing and improve the quality of their lives following surgery, these groups should involve women’s families in the treatment process.

#### A broken health system

Barriers in healthcare systems for cancer management in low-income countries and conflict areas are easy to identify, but not at all easy to rectify. The disruption and dysfunction of the health system due to repeated conflicts in such areas, the high cost of management, limited diagnostic and treatment facilities and the reduced opportunities for medical education of oncology healthcare providers are all important factors in such contexts. Some of these inequalities would have been avoided through clear guidelines for early detection aimed at shortening the pre-diagnostic journey and improving referral pathways.

The lack of adequate infrastructure for breast cancer diagnosis and treatment in Gaza has resulted in inadequate diagnosis and increased emotional and physical pain suffered by patients. These problems highlight the need to strengthen the role of female nurses to coordinate communication and treatment plans between women with breast cancer and their healthcare providers. Although there now tends to be more focus than previously on the importance of nurses in cancer care delivery in Gaza, nurses working in cancer hospitals are still general nurses rather than specialist nurses assigned to work with women with breast cancer.

Some elements of breast cancer control, such as advocacy, prevention and early detection, should also be extended to the training of community health workers, and this should be incorporated in women’s health centres. This should also include preplacement sessions to discuss methods to improve patient privacy and modesty, and gender preference of healthcare providers in the light of poor health infrastructure and limited health staff.

The establishment of well-identified breast clinics at the primary and secondary healthcare level (based on what already exists) will allow both screening of asymptomatic women aged 50 to 65 and the diagnosis of women of all ages presenting with breast signs or symptoms. The diagnosis of breast cancer at early stages would in turn reduce the cost of treatment and increase overall survival. The recent provision of health coverage for cancer services has contributed to the alleviation of socio-economic inequities among women with breast cancer in Gaza and is something women in this study were grateful for. Nevertheless, some treatment options are out of their reach, and this will probably remain the case if the country is unwilling to give up the direct provision of cancer services while remaining burdened by conflict, lack of resources and simultaneously being dependent on international funding. The priority for the coming years should be to focus on ensuring the availability, affordability and accessibility of the full range of basic and essential cancer treatments, most importantly radiotherapy, within existing cancer centres and oncology units in Gaza and ensuring sufficient fully trained staff to work there. This should include working towards the eventual plan for cancer care providers to work within a single comprehensive cancer centre with on-site access to all evidence-recommended therapies including radiotherapy.

#### Relevance of the 2023-25 conflict

The recent conflict has led to repeated internal displacements, the destruction of hospitals and clinical networks, and to border closures have sadly done little to help improve services and outcomes for these women. Consequently, cancer patients have been thrust into a state of profound anxiety, cut off from their clinical support networks and unable to seek necessary treatment elsewhere due to border closures [15]. A limited number of cancer patients have been medically evacuated to other countries, but many cancer patients have been either killed or died due to lack of access to palliative treatment. With borders closed, around 100 cancer patients per day are denied access to needed cancer care, as specialised oncology hospitals in Jerusalem and the West Bank have become inaccessible. Despite the desperate need for access to medical care, the amount of humanitarian aid currently permitted to enter Gaza is critically low. This also hampers the work of the few remaining functioning local advocacy groups, such as the Hope and Aid Organisation for Cancer Patients in Gaza. Currently, there is very little information on what is happening to women presenting with breast cancer symptoms or needing treatment in Gaza. Calls to improve access to cancer care must therefore be urgently addressed by the international cancer community.

## Ethics

The study was carried out in accordance with King’s College Data Protection Regulations (DPRF-17/18-7596), and, prior to embarking on the research, ethical clearance was obtained from the Palestinian Health Research Committee (PHRC/HC/354/18), the Palestinian Ministry of Health (223204) and the King’s College Research Ethics Committee (HR-17/18-6982). SAW explained the aim and objectives of the research study to participants who read and signed the consent form on the same day as their interview. They were assured of anonymity and a guarantee was given that participation in the study was voluntary and would have no bearing on their subsequent treatment.

## Conflicts of interest

We declare no competing interests.

## Funding

## Figures and Tables

**Table 1.** Participant’s demographic and breast cancer information.

Pseudonym		Cancer stage	Age at diagnosis	Marital status	Children	Employment status	Family history	Treatment (in order)
1.	Reem	Stage I	42	Married	4	Housewife	No	M, C, T
2.	Zeinab	Stage II	30	Married	3	Housewife	No	L, C, R
3.	Fadwa	Stage II	33	Single	0	Unemployed	Yes	L, C, T, R
4.	Ola	Stage II	33	Married	4	Housewife	No	L, C, T
5.	Fatima	Stage II	41	Married	3	Housewife	No	L, C, R, T
6.	Mona	Stage II	43	Married	3	Employed	No	L, C, T
7.	Sabreen	Stage II	43	Married	4	Housewife	No	M, C, R, T
8.	Niveen	Stage II	46	Married	3	Housewife	Yes	L, C, R
9.	Hanem	Stage II	47	Married	3	Housewife	Yes	C, L, R, T
10.	Karima	Stage II	51	Married	5	Housewife	No	M, C, T, R
11.	Nada	Stage II	54	Divorced	4	Employed	Yes	M, C, T, R, H
12.	Doha	Stage II	59	Married	4	Housewife	No	L, C, T
13.	Sara	Stage III	42	Married	3	Housewife	No	L, C, T
14.	Dina	Stage III	44	Married	3	Housewife	Yes	C, M, T, R
15.	Marwa	Stage III	45	Married	4	Housewife	No	L, M, C, T, R, H
16.	Maha	Stage III	46	Married	3	Employed	No	M, C, T, R
17.	Hamdiya	Stage III	53	Married	8	Housewife	Yes	L, C, T
18.	Jameela	Stage III	57	Married	5	Housewife	No	M, C, T, R
19.	Rasha	Stage III	58	Married	8	Housewife	No	M, C, T, R, H
20.	Nadeen	Stage IV	47	Widow	3	Housewife	Yes	C, L, T, R

Abbreviations: M = Mastectomy; L = Lumpectomy; C = Chemotherapy; R = Radiotherapy; H = Herceptin; T = Tamoxifen

**Table 2.** Conceptual framework outlining the main themes and sub-themes with relevant quotes.

Theme	Sub-theme	Relevant quotes/extracts
The diagnosis journey	(1) Why I went to see my doctor.	*“It was a lump and a swelling, and it was hard as well.”* ***Dina***, (lines: 29-31).
	(2) Initial misdiagnosis and the resulting anger caused by delays.	*“He told me: you have a cyst. Don’t be afraid and try to live with it.”* ***Jameela***, (line: 48).
	(3) Receiving, tolerating, and sharing the bad news.	*“Everything from God is good. I didn’t want to seem upset, especially in front of others.”* ***Dina***, (lines: 111-112).
**Theme**	**Sub-theme**	**Relevant quotes/extracts**
The relationship between women and the two men in their lives: the husband and the clinician	(1) Marital relationships.	*“He makes me feel bad about my illness wallah.”* ***Hanem***, (line: 605).
	(2) Being treated by a male doctor.	*“I felt it and I didn’t want to go to the doctor. I was ashamed. I did not want to go to a male doctor.”* ***Rasha***, (lines: 41-42).
	(3) Communication and privacy-related issues at the hospital.	*“I ask my doctor, but I feel like he doesn’t tell me much to be honest.”* ***Fatima****,* (line: 161). *“I’m sitting with the doctor and this man walked in and the conversation happened in front of me. So, neither I nor he had privacy.”* ***Jameela***, (lines: 521-523).
Women’s experiences of breast cancer treatment and the need for help	(1) Mastectomy or lumpectomy.	*“While they were doing the surgery, they went out to my husband and told him they wanted to do total removal because they found another lump… My husband went to pray two guidance prayers. He went back and told them not to do just partial and clean it up.”* ***Hamdiya****,* (lines:87-95).
	(2) Unmet expectations for treatment.	*“Treatment here is a bit undeveloped. The oncologist told me to take the second dose every week. Now he’s telling me that it’s not available and that I will keep taking it every three weeks.”* ***Mona***, (lines:155-159).
	(3) Coping with the disease and related psychological and financial issues.	*“My brother gave me 200 Shekels yesterday for the injections, and my husband doesn’t work.”* ***Karima***, (lines: 324-325).
